# Whole-Genome Sequencing Suggests Schizophrenia Risk Mechanisms in Humans with 22q11.2 Deletion Syndrome

**DOI:** 10.1534/g3.115.021345

**Published:** 2015-09-16

**Authors:** Daniele Merico, Mehdi Zarrei, Gregory Costain, Lucas Ogura, Babak Alipanahi, Matthew J. Gazzellone, Nancy J. Butcher, Bhooma Thiruvahindrapuram, Thomas Nalpathamkalam, Eva W. C. Chow, Danielle M. Andrade, Brendan J. Frey, Christian R. Marshall, Stephen W. Scherer, Anne S. Bassett

**Affiliations:** * The Centre for Applied Genomics and Program in Genetics and Genome Biology, The Hospital for Sick Children, Toronto, Ontario, M5G 0A4 Canada; † Clinical Genetics Research Program, Centre for Addiction and Mental Health, Toronto, Ontario, Canada; ‡ Medical Genetics Residency Training Program, University of Toronto, Ontario, M5S 1A8 Canada, University of Toronto, Toronto, Ontario, Canada; § Department of Electrical and Computer Engineering, University of Toronto, Ontario, M5S 2E4 Canada, University of Toronto, Toronto, Ontario Canada; ** Department of Psychiatry University of Toronto, Ontario, M5T 1R8 Canada, University of Toronto, Toronto, Ontario, Canada; †† Division of Neurology, Department of Medicine, University of Toronto, Ontario, M5S 1A8 Canada, University of Toronto, Toronto, Ontario, Canada; ‡‡ Epilepsy Genetics Program, Toronto Western Hospital, University Health Network and University of Toronto, Toronto, Ontario, M5T 2S8 Canada; §§ McLaughlin Centre and Department of Molecular Genetics, University of Toronto, Toronto, Ontario, M5G 0A4 Canada; *** Department of Psychiatry, and Toronto General Research Institute, University Health Network, Toronto, Ontario, Canada; ††† Campbell Family Mental Health Research Institute, Centre for Addiction and Mental Health, Toronto, Ontario, M5S 2S1 Canada; ‡‡‡ The Dalglish Family Hearts and Minds Clinic for 22q11.2 Deletion Syndrome, Toronto General Hospital, University Health Network, Toronto, Ontario, M5G 2C4 Canada

**Keywords:** 22q11 deletion syndrome, next-generation sequencing, genetic architecture, copy number variation, microRNA, *DGCR8*, schizophrenia, noncoding RNA, lincRNA, *FMR1*, synapse, connectivity, postsynaptic density, polygenic risk score, *ABLIM1*, *BSN*, *DIP2A*, *EXOC4*, *ITM2C*, *MYH9*, *MYH10*, *PCNT*, *PTPRG*, *SLITRK2*, *ZDHHC5*

## Abstract

Chromosome 22q11.2 microdeletions impart a high but incomplete risk for schizophrenia. Possible mechanisms include genome-wide effects of *DGCR8* haploinsufficiency. In a proof-of-principle study to assess the power of this model, we used high-quality, whole-genome sequencing of nine individuals with 22q11.2 deletions and extreme phenotypes (schizophrenia, or no psychotic disorder at age >50 years). The schizophrenia group had a greater burden of rare, damaging variants impacting protein-coding neurofunctional genes, including genes involved in neuron projection (nominal *P* = 0.02, joint burden of three variant types). Variants in the intact 22q11.2 region were not major contributors. Restricting to genes affected by a *DGCR8* mechanism tended to amplify between-group differences. Damaging variants in highly conserved long intergenic noncoding RNA genes also were enriched in the schizophrenia group (nominal *P* = 0.04). The findings support the 22q11.2 deletion model as a threshold-lowering first hit for schizophrenia risk. If applied to a larger and thus better-powered cohort, this appears to be a promising approach to identify genome-wide rare variants in coding and noncoding sequence that perturb gene networks relevant to idiopathic schizophrenia. Similarly designed studies exploiting genetic models may prove useful to help delineate the genetic architecture of other complex phenotypes.

Schizophrenia is a complex neuropsychiatric disease with prominent genetic heterogeneity. The established molecular genetic risk factors of largest effect are rare copy number variations (CNVs), especially 22q11.2 deletions (Kirov *et al.* 2012; Costain *et al.* 2013; Stankiewicz and Lupski 2010; Lowther *et al.* 2015; Bassett *et al.* 2010; Hochstenbach *et al.* 2011; Costain and Bassett 2012; Rees *et al.* 2014). Whole-exome sequencing (WES) studies indicate that rare coding sequence variants also contribute to schizophrenia (Girard *et al.* 2011; Xu *et al.* 2012; Need *et al.* 2012; Gulsuner *et al.* 2013; Timms *et al.* 2013; Fromer *et al.* 2014; Purcell *et al.* 2014; McCarthy *et al.* 2014; Guipponi *et al.* 2014). Rare variants that disrupt mechanisms regulating expression of protein-coding genes are likely to be part of the genetic architecture of schizophrenia as well (Morrow 2015; Geaghan and Cairns 2014; Forstner *et al.* 2013; Beveridge and Cairns 2012; Moreau *et al.* 2011; Xu *et al.* 2010; Warnica *et al.* 2015). These variants usually alter noncoding RNA gene exons or splicing and transcription regulatory motifs that typically reside outside of protein-coding exons. For this reason, the majority of these variants are detectable only by the use of whole-genome sequencing (WGS).

Extensive research efforts have focused on understanding the contribution of common variation to schizophrenia risk. The most recent large-scale study successfully identified more than 100 genome-wide significant loci, although with very modest effect size and often obscure molecular mechanisms (Schizophrenia Working Group of the Psychiatric Genomics Consortium 2014). A polygenic risk score, based on the additive contribution of many weakly associated variants (International Schizophrenia Consortium *et al.* 2009), has been used successfully to maximize the fraction of schizophrenia risk explained by common variation (Schizophrenia Working Group of the Psychiatric Genomics Consortium 2014). Pathway-level methods also have been investigated to identify commonalities among many different contributing variants, rare or common (Kirov *et al.* 2012; Costain *et al.* 2013; Fromer *et al.* 2014; Purcell *et al.* 2014; Warnica *et al.* 2015; Pathway Analysis Subgroup of Psychiatric Genomics Consortium Network 2015).

Even in 22q11.2 deletion syndrome (22q11.2DS), where the recurrent 22q11.2 deletion imparts a 25% risk of developing schizophrenia (Fung *et al.* 2010; Schneider *et al.* 2014), there remain undiscovered determinants of expression. One proposed mechanism involves genome-wide microRNA (miRNA) dysregulation related to haploinsufficiency of the *DGCR8* gene that lies within the 22q11.2 deletion region (Stark *et al.* 2008; Forstner *et al.* 2013; Schofield *et al.* 2011; Brzustowicz and Bassett 2012; Merico *et al.* 2014). In individuals with 22q11.2 deletions this haploinsufficiency could increase susceptibility to the effects of protein-coding mutations that otherwise may be tolerated, including those in genes that are involved in schizophrenia in the general population (Brzustowicz and Bassett 2012).

In this initial proof-of-principle study, we hypothesized that the 22q11.2 deletion would provide enhanced power to investigate biologically plausible mechanisms for schizophrenia. We used high-quality, WGS of nine individuals with 22q11.2 deletions and extreme phenotypes (schizophrenia, or no psychotic disorder at age >50 years), followed by a comprehensive annotation and prioritization of rare variants impacting coding and non-coding sequence (Yuen *et al.* 2015). To maximize statistical power, we investigated rare variant burden for gene-sets with higher *a priori* likelihood of contributing to schizophrenia risk. We additionally investigated common variant contribution using a polygenic risk score model.

We found evidence for rare variants outside the 22q11.2 region perturbing gene networks relevant to idiopathic schizophrenia, for a *DGCR8*/miRNA-related mechanism, for other noncoding sequence variants, and for a polygenic risk contribution, and predicted that maximal statistical power can be achieved with attainable sample sizes of this genetic model.

## Methods and Materials

### Subjects

From a cohort of Canadian adults with 22q11.2DS (Bassett *et al.* 2003, 2008; Brzustowicz and Bassett 2012; Cheung *et al.* 2014; Fung *et al.* 2010; Schneider *et al.* 2014; Vorstman *et al.* 2013; Swaby *et al.* 2011; Butcher *et al.* 2012, 2013, 2015), we selected nine unrelated individuals of European descent (Table 1), based on availability of high quality genomic DNA for WGS and phenotypic information consistent with the extreme phenotype design: six (SCZ1-SCZ6) met DSM-IV criteria for schizophrenia or schizoaffective disorder (Bassett *et al.* 2003) and three (NP1-NP3) had no psychotic disorder at age >50 years (Table 1). Deep phenotyping included direct clinical assessments at multiple time points and review of lifetime medical records, with the use of our established methods (Fung *et al.* 2010; Vorstman *et al.* 2013; Swaby *et al.* 2011; Butcher *et al.* 2012, 2013; Cheung *et al.* 2014; Bassett *et al.* 2003). The six subjects with schizophrenia had no other single major feature of 22q11.2DS in common (Table 1). All participants provided written informed consent, and the study was approved by local research ethics boards.

**Table 1:** Characteristics of nine adults of European ancestry with 22q11.2 deletions and whole-genome sequencing data

Case Identifier	Schizophrenia						Nonpsychotic		
	SCZ1	SCZ2	SCZ3	SCZ4	SCZ5	SCZ6	NP1	NP2	NP3
Schizophrenia phenotype and risk factors									
Age at last follow-up or at death (yr)	56	58	38	48	44	21	61	53	52
Age at onset of psychosis (yr)	17	22	15	18	21	12	−	−	−
Treatment-resistance*^a^*	No	No	No	Yes	Yes	No	−	−	−
Substance abuse*^b^*	No	No	No	No	No	No	No	No	No
Developmental brain anomaly*^c^*	No	No	No	Yes	Yes	Yes	No	No	No
Family history of schizophrenia*^d^*	No	No	No	No	Yes	No	No*^e^*	No*^e^*	No
Additional demographic, genotypic, and phenotypic features									
Sex	Female	Male	Male	Female	Male	Female	Male	Male	Female
22q11.2 deletion type*^f^*	Nested	Typical	Typical	Typical	Typical	Typical	Typical	Typical	Typical
* De novo* 22q11.2 deletion*^f^*	Probable	Probable	Yes	Yes	Probable	Probable	Probable	Yes	Probable
Major feature of 22q11.2DS*^g^*									
Congenital heart disease*^h^*	No	Yes	No	No	Yes	Yes	No	No	Yes
Cleft palate and/or velopharyngeal insufficiency	No	No	No	Yes	No	Yes	Yes	No	Yes
Intellectual disability*^i^*	Borderline	No	Mild	Borderline	Borderline	Mild	No	Borderline	Borderline
Seizures	Single	No	Multiple	Multiple	Single	Multiple	No	No	No
Major mood or anxiety disorder*^j^*	No	No	No	No	No	Yes	No	Yes	Yes
Parkinson’s disease*^k^*	Yes	Yes	No	No	No	No	Yes	No	No

a Requiring trial of clozapine (Butcher *et al.* 2015).

b Nicotine excepted.

c On neuroimaging studies (Andrade *et al.* 2013) and/or postmortem examination (Kiehl *et al.* 2009; Butcher *et al.* 2014).

d Schizophrenia or schizoaffective disorder in a first-degree relative without a 22q11.2 deletion.

e Both have adult offspring who inherited the 22q11.2 deletion and also do not have a psychotic disorder.

f Although not part of the study design, *de novo* 22q11.2 deletions are typical in 22q11.2DS; breakpoints for typical (∼2.6−3.0 Mb; ∼90% of 22q11.2DS) and proximal nested (∼1.3−1.5 Mb; ∼5% of 22q11.2DS) 22q11.2 deletions, and *de novo* status, are defined in Bassett *et al.* (2008).

g As described in Fung *et al.* (2015).

h Tetralogy of Fallot (SCZ2), atrial septal defect and ventricular septal defect (SCZ5, NP3), ventricular septal defect (SCZ6).

i As assessed in Butcher *et al.* (2012).

j Obsessive compulsive disorder (SCZ6), generalized anxiety disorder (NP2, NP3).

k Diagnostic details and additional phenotype data for the three subjects with Parkinson's disease are reported elsewhere (Butcher *et al.* 2013).

### WGS approach and methods

We submitted a high-quality genomic DNA sample from each subject to Complete Genomics for WGS (Drmanac *et al.* 2010; Carnevali *et al.* 2012). Mean genome coverage per sample was 98.95% (98.81–99.10%) at depth ≥5X and 97.65% (97.30–98.15%) at depth ≥10X, relative to the hg19 human genome reference sequence. In particular, 94.4% and 72.3% of the exome was covered with at least 20X and 40X sequence depth, respectively. Complete Genomics data for each nucleotide position, supplemented by in-house protocols, provided stringent quality filters. For this study, we used only high-quality variants (those with high confidence scores). Variants were then annotated with a custom pipeline based on the ANNOVAR (November 2014) software tool (Wang *et al.* 2010). We defined rare variants as those at <1% of the alternate allele frequency (minor allele frequency = 0.01) threshold in each of three standard [1000 Genomes (1000 Genomes Project Consortium *et al.* 2012), National Heart, Lung, and Blood Institute Exome Sequencing Project (Fu *et al.* 2013), Exome Aggregation Consortium (http://exac.broadinstitute.org/)], and two in-house, platform-matched databases. Details of all WGS-related laboratory and data interpretation/bioinformatics methods used are provided in the Supporting Information, Figure S1 and File S1.

### Rare variant burden analyses for coding genes

We considered the possible impact of accumulated deleterious variants affecting protein-coding genes under a haploinsufficiency model, excluding variants in the intact chromosome 22q11.2 region and on the X chromosome, which were examined separately. These variants comprised three categories: loss of function (LoF) variants (stop-gain/nonsense, frameshift, and core splice site), damaging missense variants (predicted to be deleterious per five of seven standard tools), and splicing regulatory variants that (negatively) affect exon inclusion; the latter include intronic variants that are further away from core splice site (LoF) variants (Xiong *et al.* 2015). First, we tested “neurofunctional” gene-sets (*i.e.*, affecting brain-related functions most likely to be important to schizophrenia expression), separating each variant category (LoF, missense, splicing regulatory). Gene-sets with nominally significant burden for at least one variant category (*P* < 0.10 for LoF and splicing regulatory, and *P* < 0.05 for missense variants) were then tested for the joint burden of the three variant categories with a multivariate, two-sample Hotelling’s T-Square test (Hotelling 1931). To investigate a *DGCR8*/miRNA mechanism, we used the same gene-sets but restricted to those genes predicted to be affected by *DGCR8* haploinsufficiency (Stark *et al.* 2008). To estimate the burden effect size, we calculated the between-group ratio of the mean absolute variant count (Hu *et al.* 2009). For gene-set burden power calculations, we selected four representative gene-sets showing enrichment for one or more of the variant categories, and used Cohen’s d to express the effect size estimates.

### Copy number variation

We evaluated CNVs and other structural variants (SVs) by using a previously established annotation and prioritization process (Yuen *et al.* 2015). All subjects were confirmed to have 22q11.2 deletions (Table 1). Of the remaining variants, only rare CNVs and SVs that overlapped at least one coding gene exon of a RefSeq gene with known neuronal function were considered in this study.

### Rare variant burden analyses for noncoding RNA genes

We considered two main types of noncoding RNA variants: miRNA derived from mirBase v20 (Griffiths-Jones 2004) and long intergenic noncoding RNA (lincRNA) derived from the Broad catalog (Cabili *et al.* 2011). We tested the burden of high-quality, rare variants prioritized based on regional and nucleotide-level genomic conservation.

### Common variant polygenic risk score

We obtained the list of 102,636 SNPs used by the Psychiatric Genomics Consortium to define a risk score for schizophrenia, together with the original nominal association p-values and odds ratios (International Schizophrenia Consortium *et al.* 2009; Schizophrenia Working Group of the Psychiatric Genomics Consortium 2014). These SNPs were mapped to hg19 coordinates and intersected with the WGS data for our cohort; in particular, WGS variants were matched to risk score SNPs by coordinates and alleles, whereas WGS reference intervals (*i.e.*, identical to the human reference sequence) were matched by coordinate overlap. A total of 88,301 SNPs was successfully mapped to variants passing quality filters, or reference intervals, in all nine genomes in this study. Allele counts were computed as the number of alleles matching to the allele used for association analysis (possible values: 0, 1, 2). The SNP-wise risk score was then calculated as the product of this allele count and the log(odds-ratio) (Schizophrenia Working Group of the Psychiatric Genomics Consortium 2014). Using nominal p-value thresholds ≤0.001 and ≤0.0001, as well as one more stringent (≤0.00001), and p-values >0.9 and >0.5 as negative controls (Schizophrenia Working Group of the Psychiatric Genomics Consortium 2014), the polygenic risk scores for each 22q11.2DS subject were then calculated as the sum of all respective SNP-wise risk scores (International Schizophrenia Consortium *et al.* 2009). Differences between schizophrenia and no-psychosis groups were tested using a one-sided *t*-test and a Wilcoxon test. We also calculated the percentage of correctly predicted schizophrenia and no-psychosis subjects at different risk score values, and reported the maximum value as a point-estimate of separation between the two groups.

### Data availability

Supporting Information contains detailed descriptions of all supplemental files. Figure S1 contains selected gene-sets with a higher burden in subjects with schizophrenia. Figure S2 contains distribution boxplots of subjects' polygenic risk scores for the schizophrenia and nonpsychotic groups. Table S1 contains high quality, rare coding variants. Table S2 contains source and size of gene-sets used in the burden analyses. Table S3 contains details of burden analyses for each type of variants. Table S4 contains most recurrent splicing regulatory predictive features detected in this study. Table S5 contains details of power calculations. Table S6 contains details of burden analyses for lincRNA. Table S7 contains details of lincRNA with high quality, rare variants and miRNA with high quality rare variants.

## Results

Details of subjects with 22q11.2DS are in Table 1. Subjects had an average of 13.8 and 94.3 high-quality, rare variants disrupting coding genes (LoF and missense categories, respectively), with similar findings for both the schizophrenia and non-psychotic groups (Table S3). There were few additional variants in the intact chromosome 22q11.2 region (Table 2).

**Table 2:** Selected brain function related gene-set results for rare single nucleotide variants

Brain Function Related Gene-Set	Genes Disrupted in SCZ Cases			Mean Number of Variants per Subject*^a^*		*P**^b^*	Estimated Effect Size (Ratio of Means)
	Per Gene-Set	In Neuron Projection Gene-Set					
	Total n	n	(%)	SCZ	NP		
Damaging missense variants							
Neuron projection (GO)	53	53	(100)	9.00	5.00	0.009	1.80
Restricted to *DGCR8*-related genes*^c^*	16	16	(100)	2.67	0.67	0.025	4.00
Synaptic pathways (KEGG)	15	3	(20)	2.50	1.00	0.053	2.50
Restricted to *DGCR8*-related genes*^d^*	7	0	(0)	1.17	0	0.005	nc
GABAergic synapse (KEGG)*^e^*	7	1	(14)	1.17	0	0.015	nc
Restricted to *DGCR8*-related genes	3	0	(0)	0.50	0	0.039	nc
Cholinergic synapse (KEGG)*^e^*	6	2	(33)	1.00	0.33	0.152	3.00
Restricted to *DGCR8*-related genes	3	0	(0)	0.50	0	0.038	nc
Abnormal sensory system (MGI)	58	13	(22)	10.17	8.00	0.029	1.27
Restricted to *DGCR8*-related genes	19	7	(37)	3.33	1.00	0.024	3.33
Neural function or pathway, union, stringent (GO, KEGG, NCI, Reactome)	65	49	(75)	11.00	6.33	0.026	1.74
Restricted to *DGCR8*-related genes	21	13	(62)	3.50	1.67	0.023	2.10
Nervous system abnormality, autosomal dominant or X-linked (HPO)	31	9	(29)	5.50	2.67	0.018	2.06
Higher mental function abnormality, autosomal dominant or X-linked (HPO)	5	2	(40)	0.83	0	0.019	nc
Nervous signal transmission (GO)	26	12	(46)	4.33	2.00	0.049	2.17
Schizophrenia risk candidate genes (six WES studies)*^f^*	45	14	(31)	7.50	7.67	0.573	0.98
Restricted to *DGCR8*-related genes*^g^*	11	7	(64)	1.83	1.00	0.020	1.83
Loss of function variants							
Post-synaptic density (Bayes *et al.* 2011)*^h^*	8	2	(25)	1.33	0.33	0.128	4.00
Restricted to *DGCR8*-related genes	4	2	(50)	0.67	0	0.047	nc
Abnormal sensory system (MGI)	6	2	(33)	1.00	0	0.013	nc
Splicing regulatory variants							
* FMR1* targets (Ascano *et al.* 2012)*^i^*	7	1	(14)	1.17	0	0.018	nc

SCZ, schizophrenia subgroup of 22q11.2DS subjects; NP, nonpsychotic subgroup of 22q11.2DS subjects; GO, Gene Ontology; KEGG, Kyoto Encyclopedia of Genes and Genomes; nc, not calculable (based on no variants present in the non-psychotic group); MGI, Mouse Genome Informatics; NCI, National Cancer Institute; HPO, Human Phenotype Ontology.

Gene-sets portrayed are all those with nominal p value <0.05 before and/or after restriction to *DGCR8* related genes, with <2000 genes in gene-set. All high-quality, rare variants contributing to the results are reported in Table S1. For source and total size of each gene-set, see Table S2; for total gene overlap between gene-sets, see Table S2; for burden analysis results for all gene-sets, see Table S3.

Genes implicated by variants in the schizophrenia group (genes in the Neuron projection (GO) gene-set, thus contributing to the Hotelling analysis results, are in bold font):

a Variants in the intact chromosome 22q11.2 region and on the X chromosome were *a priori* excluded from the burden analyses. In total, there were three rare damaging missense variants in the 22q11.2 region [involving the genes *DGCR2* (NP1), *GNB1L* (NP3), and *TRMT2A* (SCZ1)], and eight rare, damaging SNVs (seven missense, one LoF) on the X chromosome [involving the genes *COL4A6*​ (NP3), *JADE3* (SCZ3), *KLHL15* (SCZ6), *LRCH2*​ (SCZ6), *PFKFB1* (NP3), *SLC25A43* (NP3; LoF variant), *SLITRK2* (SCZ2), and *TBC1D8B* (NP1)]. Only two would have contributed to the results in this table. *DGCR2* is in the Schizophrenia risk candidate genes (6 WES studies) gene-set and *SLITRK2* (Piton *et al.* 2011) is in the Neuron projection (GO) gene-set. Neither gene is in the *DGCR8*-related gene-set.

b Nominal (one-sided t-test) p value using percent values (means corrected for total number of all variants of that type per subject, *e.g.*, all missense variants)

c ***ACTN4**, **ANK1**, **ARHGEF7**, **BSN**, **COL3A1**, **COL9A1**, **ITM2C**, **MAP1B**, **MAP2**, **MYH10**, **MYH9**, **PTPRG**, **SLITRK6**, **STIM1**, **TGFB2**, **ZDHHC5***.

d *ADCY3, KCNQ5, PLCL1, PLD1, PPP2R3A, PRKACB, SLC1A7*.

e Gene-set having 100% overlap with Synaptic pathways (KEGG) gene-set.

f WES, whole-exome sequencing studies: (Girard *et al.* 2011; Xu *et al.* 2012; Gulsuner *et al.* 2013; Fromer *et al.* 2014; McCarthy *et al.* 2014; Guipponi *et al.* 2014).

g ***ANK1**, **COL3A1**, EPHA2, **ITM2C**, KCNQ5, **MYH10**, **MYH9**, NUP210, **PTPRG**, UTRN, **ZDHHC5***.

h ***ABLIM1**, DDX6, **EXOC4**, FARSB, ITSN2, PDE1A, TAGLN2, UBR4* (***ABLIM1***, ***EXOC4***, *ITSN2*, *TAGLN2* = with *DGCR8* restriction)

i *AP1B1, **AP3D1**, DNM2, RYR2, SETDB2, VPRBP, ZNF107*.

### Burden of rare variants impacting neurofunctional protein-coding genes

Table 2 shows all gene-sets with <2000 protein-coding genes and nominally significant (*P* < 0.05, schizophrenia > nonpsychotic group) burden for rare deleterious variants. Only neurofunctional gene-sets met these criteria. On testing burden jointly for all three variant categories, only the Neuron projection [Gene Ontology (GO)] gene-set was significant (Hotelling’s T-Square *P* = 0.02). Table 2 shows the overlap between this and the other neurofunctional gene-sets for genes implicated in the schizophrenia group.

As predicted by a multiple within-person rare variant hypothesis for schizophrenia (Costain *et al.* 2013; Merico *et al.* 2014), there were several variants per subject involving these neurofunctional gene-sets. There were no significant between-group differences for larger gene-sets, or even all brain-expressed variants (Table S3). The findings support an approach focused on high-quality variants and gene-sets of neurofunctional relevance, even in this small sample. Table S1, Table S2, Table S3, and Table S4 show details for all high-quality, rare variants, gene-sets used, and burden analysis results.

We used the data available for the three variant types to perform power calculations for the Neuron projection (GO) gene-set burden test and three other gene-sets (Table S5). For N = 100 subjects per group, power for the GO gene-set was >0.99 for damaging missense variants and for LoF variants, and >0.94 for splicing regulatory variants (Cohen’s d effect sizes: 1.90, 0.88, 0.55, respectively; the effect size estimates are based on the nine genomes presented in this study). For the Post-synaptic density (Bayes *et al.* 2011) gene-set, power was >0.99 for LoF variants and for splicing regulatory variants. Other results showing power >0.99 are in Table S5.

### Support for the *DGCR8*/miRNA hypothesis

Consistent with a miRNA hypothesis for schizophrenia, restricting to genes predicted to be affected by *DGCR8* haploinsufficiency (Stark *et al.* 2008; Merico *et al.* 2014) tended to increase estimated effect sizes (Table 2 and Figure S1), despite the decrease in number of variants per subject. For missense variants, these gene-sets included Neuron projection (GO) and Synaptic pathways (Kyoto Encyclopedia of Genes and Genomes KEGG), with no overlap of the genes involved between these gene-sets. For LoF variants, the Post-synaptic density (Bayes *et al.* 2011) gene-set was implicated (Table 2). Restricting to *DGCR8*-related genes did not tend to increase effect size for splicing regulatory variants (Figure S1). Notably, applying the *DGCR8*-related gene filter revealed nominally significant burden in 22q11.2DS-schizophrenia for rare damaging missense variants using a gene-set from idiopathic schizophrenia WES studies (*de novo* nonsynonymous variants) (Girard *et al.* 2011; Xu *et al.* 2012; Gulsuner *et al.* 2013; Fromer *et al.* 2014; McCarthy *et al.* 2014; Guipponi *et al.* 2014). Several of the genes involved overlapped those in the Neuron projection (GO) gene-set (Table 2).

### Rare CNV disrupting candidate genes for schizophrenia

Similar to our previous study focusing on CNV >10 kb in size (Bassett *et al.* 2008), we interrogated the genome outside of the 22q11.2 region for additional rare CNVs and SVs. In one individual with schizophrenia (SCZ4), we identified and confirmed via quantitative polymerase chain reaction a rare maternally inherited 84-kb deletion at 21q22.3. This CNV disrupts exons of the genes *PCNT* and *DIP2A*, the latter gene implicating *DGCR8* and *FMR1* interactome mechanisms (Stark *et al.* 2008; Darnell *et al.* 2011).

### Rare variants disrupting noncoding RNA genes

There were multiple rare variants outside of protein-coding genes, on average involving 2.0 and 1.3 lincRNA genes per subject in the schizophrenia and nonpsychotic groups, respectively (Table S6). Restricting to highly conserved (top 10%) lincRNAs, the burden was greater in the schizophrenia group (mean 1.3 *vs.* 0; nominal *P* = 0.039) (Table S6). However, perhaps related to their small size, miRNA genes contained few rare variants, even after broadening the rarity definition to <5%, preventing statistical testing of burden (Table S7).

### Schizophrenia polygenic risk score

Use of the selected schizophrenia-associated SNPs [at nominal p-value thresholds ≤0.001 and ≤0.0001 from the Psychiatric Consortium Study (International Schizophrenia Consortium *et al.* 2009; Schizophrenia Working Group Of The Psychiatric Genomics Consortium 2014)] resulted in greater polygenic risk scores in the 22q11.2DS schizophrenia than in the nonpsychotic group (for the two thresholds, respectively, based on 2866 and 1059 SNPs: means: 0.00798 *vs.* −2.482, 0.601 *vs.* −1.238; *t*-test p-values: *P* = 0.094, *P* = 0.064; Wilcoxon test p-values: *P* = 0.083, *P* = 0.190; maximum correctly predicted percentages: 83%, 75%) (Figure S2). These trends did not reach our definition of statistical significance, however. The Wilcoxon and *t*-test p-values were greater (0.136 and 0.274, respectively) using association threshold *P* ≤ 0.00001 (451 SNPs). As expected, almost no difference (Wilcoxon and *t*-test *P* = 0.32−0.80) was observed for negative control SNPs (18,675 and 1976 SNPs at association p-value thresholds *P* > 0.5 and *P* > 0.9, respectively).

## Discussion

Historically, the psychiatric genetics field has not used a genetic model or a functionally and mechanistically driven approach as a means to evaluate germline genetic variation in schizophrenia. We demonstrate the potential success of exploiting the enhanced homogeneity and thus power of a genomic disorder (rare and highly penetrant CNV) to investigate expression of a major associated disease phenotype. This study of schizophrenia in 22q11.2DS had two primary goals: (i) to demonstrate an effective approach to analyzing and interpreting WGS data, based on previous success in autism (Yuen *et al.* 2015), and (ii) to identify and prioritize testable, biologically plausible hypotheses for further investigation in a larger sample. That there were findings reaching our definition of nominal statistical significance was unexpected in this small sample. The results demonstrate the power and generalizability of 22q11.2DS as a model for understanding the genetic architecture of idiopathic schizophrenia, and provide support for multiple rare variants within individuals and a miRNA-related mechanism. These are concepts with previous evidence (Girirajan *et al.* 2012; Warnica *et al.* 2015).

By definition, individuals with 22q11.2DS have a 22q11.2 deletion, thus identifying additional rare variants would support a multiple rare variant hypothesis for schizophrenia at the individual level. The findings of this study indicate that this is likely to involve not only exonic variants, as expected, but also variants in regulatory regions and noncoding RNA genes typically not detectable by WES technologies. In the subgroup of individuals with schizophrenia, there was evidence for enrichment of damaging variants in highly conserved lincRNA (nonprotein-coding) genes, and of certain splicing regulatory variants that affect protein-coding genes. Although functional characterization of lincRNAs is limited as yet, the strategy used here may help to identify lincRNAs that contribute to schizophrenia. lincRNAs are involved in epigenetic mechanisms including chromatin binding, and in splicing processes (Barry *et al.* 2014; Quek *et al.* 2015; Derrien *et al.* 2012; Moran *et al.* 2012). Interestingly, the gene-set most affected by splicing regulatory variants in this study implicates mRNA targets of *FMR1*, and thus post-transcriptional regulation of gene expression, including that involved in neuronal development and synaptic plasticity (Pinto *et al.* 2014; Suhl *et al.* 2014).

The burden analyses of the coding sequence variants further demonstrated the effectiveness of the approach used to analyze and interpret WGS data. In the subgroup of individuals with schizophrenia, using biologically informed filters revealed a greater burden of damaging variants affecting protein-coding genes involved in neuron projection (axonal and dendritic development), a gene-set previously implicated in schizophrenia using other approaches (Costain *et al.* 2013; Merico *et al.* 2014).

Restricting to genes affected by *DGCR8* haploinsufficiency tended to increase effect sizes for neurofunctionally relevant gene-sets. The findings thus provide further support for a miRNA hypothesis for schizophrenia and the utility of 22q11.2DS as a model for this mechanism (Warnica *et al.* 2015; Merico *et al.* 2014; Morrow 2015; Geaghan and Cairns 2014; Moreau *et al.* 2011). The 22q11.2 deletion appears to act as a threshold-lowering first hit, likely in part related to haploinsufficiency of gene *DGCR8* and its effects on miRNA buffering, to reveal effects of rare variants elsewhere in the genome (Stark *et al.* 2008; Forstner *et al.* 2013; Schofield *et al.* 2011; Brzustowicz and Bassett 2012; Merico *et al.* 2014). This included variants, present in each of the 22q11.2DS subjects with schizophrenia, in genes previously reported for idiopathic schizophrenia in WES studies (Girard *et al.* 2011; Xu *et al.* 2012; Gulsuner *et al.* 2013; Fromer *et al.* 2014; McCarthy *et al.* 2014; Guipponi *et al.* 2014).

Lastly, the polygenic risk score appears informative for the nine 22q11.2DS genomes, although probably because of the small sample size, the results do not achieve significance. Future studies with sufficient power to jointly model rare variant burden and common variant polygenic risk score would be useful, and could determine whether restricting the polygenic risk score SNPs to those implicating genes from neurofunctional gene-sets would amplify between-group differences.

### Advantages and limitations

Although this initial study produced several nominally significant results, there was no correction for multiple comparisons. Part of our *a priori* design was that any findings would require replication with the use of larger samples. The estimates of effect size and power indicate that feasible sample sizes of individuals with 22q11.2DS will allow such replication, using a comparable design and approach. Our analytic strategy was designed to minimize both false-positive and false-negative results. In the absence of between-group differences in total burden of rare variants, individual false-positive results would be expected to affect both groups equally. All individuals would be expected to harbor multiple rare variants involved in neurofunctional gene-sets. Among genes in neurofunctional gene-sets, a specific subset may eventually be identified to make a greater contribution to the expression of schizophrenia in all, or in certain subforms, of the disorder. These could include genes where there are individual, rare damaging variants with large effect. Nonetheless, we expect a substantial level of polygenicity, as suggested by rare variant studies of schizophrenia and other neuropsychiatric disorders such as autism, as well as by the paucity of linkage findings for schizophrenia (Kirov *et al.* 2012; Costain *et al.* 2013; Girard *et al.* 2011; Xu *et al.* 2012; Need *et al.* 2012; Gulsuner *et al.* 2013; Timms *et al.* 2013; Fromer *et al.* 2014; Purcell *et al.* 2014; McCarthy *et al.* 2014; Guipponi *et al.* 2014; Pinto *et al.* 2014; Yuen *et al.* 2015).

As for the largest WES study in schizophrenia to date (Purcell *et al.* 2014), and our previous CNV studies (Pinto *et al.* 2014; Costain *et al.* 2013; Silversides *et al.* 2012), increased stringency of methods and approach, including quality, rarity, and deleteriousness of variants, generally strengthened the findings. Individual sequence variants were not validated by the use of a second method. Using a comparable WGS analytic pipeline, we found that greater than 90% of rare *de novo* SNVs were validated in a study of autism (Yuen *et al.* 2015); we expect this to be the minimum validation rate in this study. We would not restrict future studies to *de novo* variants, however, because most rare variants are inherited and may have enhanced impact in the context of a 22q11.2 deletion (Stark *et al.* 2008; Forstner *et al.* 2013; Schofield *et al.* 2011; Brzustowicz and Bassett 2012; Merico *et al.* 2014). For variants in nongenic regulatory regions, WGS is essential for detection with clear advantages over WES studies, including one involving two individuals with 22q11.2DS (Balan *et al.* 2014). Increasingly sophisticated methods offer promise for improved *in silico* evaluation of all variant types. Proof that a variant is causal, however, requires a laboratory-based functional analysis.

The size of this study limited the ability to explore interacting factors or to study other hypotheses of interest, including the role of individual common variants and nongenetic factors. Previous studies of SNPs on the intact chromosome 22q11.2 have shown conflicting or negative results, however reduced gene dosage of neurofunctional genes within the 22q11.2 region may contribute to schizophrenia risk (Philip and Bassett 2011; Arinami 2006; Prasad *et al.* 2008; Karayiorgou *et al.* 2010). Although we did not identify rare variants disrupting candidate genes in this region in individuals with schizophrenia, rare phenotypes may be attributable to unmasking of such variants (McDonald-McGinn *et al.* 2013). Our WGS approach could be generalizable to other phenotypes associated with 22q11.2DS, and help explain variable expression and incomplete penetrance in other genomic disorders. Also, although additional rare CNVs overlapping exons may play a minor role, consistent with previous results (Bassett *et al.* 2008; Williams *et al.* 2013), methods for studying small CNVs require refining. Nonetheless, the initial evidence of multiple rare variants within an individual, coupled with suggestive findings for polygenic common variant risk, provided by this study is consistent with a longstanding threshold model of schizophrenia.

Early studies of 22q11.2 deletions foreshadowed a more general role for rare CNV in understanding the global genetic architecture of schizophrenia in the population (Kirov *et al.* 2012; Costain *et al.* 2013; Stankiewicz and Lupski 2010; Lowther *et al.* 2015; Bassett *et al.* 2010; Hochstenbach *et al.* 2011; Costain and Bassett 2012; Zarrei *et al.* 2015; Rees *et al.* 2014). Here, results from this study indicate that, for the individual, to uncover the symphony of variants that increase the likelihood of the expression of schizophrenia, researchers can take advantage of the fact that a highly penetrant rare CNV like the 22q11.2 deletion represents an incomplete part of the genetic architecture. This study design may lead to the discovery of novel additional pathways from genotype to phenotype in schizophrenia. Findings from this study provide support for a tractable, mechanistically and functionally based approach for evaluating the myriad rare coding-sequence variants identified by WGS and their potential role along with common variant risk in individual genetic architecture, the importance of evaluating variants in noncoding sequence, and the enhanced power to identify relevant rare variants that may be afforded by a more genetically homogeneous sample. The main genetic sharing between unrelated individuals with schizophrenia appears to be at the pathway/mechanistic level, thus a research design with this focus promises robust findings. Studies that also combine common variant risk with a rare damaging variant gene-set burden model may be of particular interest.

## 

## Supplementary Material

Supporting Information
