# Supplementary material for: Whole-Genome Sequencing Suggests Schizophrenia Risk Mechanisms in Humans with 22q11.2 Deletion Syndrome
Source: G3 (Bethesda). 2015 Sep 16;5(11):2453–61. doi: 10.1534/g3.115.021345 (PMC4632064; doi:10.1534/g3.115.021345)
Supplement: Supporting Information [file supp_5_11_2453__index.html]

Whole-Genome Sequencing Suggests Schizophrenia Risk Mechanisms in Humans with 22q11.2 Deletion Syndrome — Supporting Information 

# Whole-Genome Sequencing Suggests Schizophrenia Risk Mechanisms in Humans with 22q11.2 Deletion Syndrome

## Supporting Information for Merico *et al.*, 2015

**Files in this Data Supplement:**

- Supporting Information - Figures S1-S2, File S1, and Tables S1-S7 (PDF, 1 MB)
- Figure S1 - Selected gene-sets with a higher burden of (a) damaging missense, (b) loss of function (LoF), and (c) splicing regulatory variants in those with 22q11.2DS and schizophrenia (SCZ1-SCZ6) compared with those with 22q11.2DS and no psychotic disorder (NP1-NP3). (PDF, 638 KB)
- Figure S2 - Distribution boxplots of subjects' polygenic risk scores for the schizophrenia (brown outline) and non-psychotic (blue outline) groups, using the SNPs at association p-value cutoff of 0.001 (10^-3) and 0.0001 (10^-4). (PDF, 589 KB)
- File S1 - Supplemental Methods (PDF, 956 KB)
- Table S1 - High quality, rare (<1% frequency) coding variants in subjects with 22q11.2DS used in the gene-set burden analyses. (.xlsx, 1 MB)
- Table S2 - Source, size, and pair-wise intersection of gene-sets used in the burden analyses, by number of genes and percent overlap. (.xls, 295 KB)
- Table S3 - Details of burden analyses for each of damaging missense, loss of function, and splicing regulatory variant categories, comparing subjects with 22q11.2DS and schizophrenia to subjects with 22q11.2DS and no psychotic disorder. (.xlsx, 91 KB)
- Table S4 - Most recurrent splicing regulatory predictive features detected in this study (see Supplemental Methods and (Xiong *et al.* 2015) for details). (.xls, 45 KB)
- Table S5 - Details of power calculation for enrichment in selected gene-sets based on the results of the current study. (.xls, 38 KB)
- Table S6 - Details of burden analyses for lincRNAs with rare conserved variants. (.xlsx, 14 KB)
- Table S7 - Details of lincRNAs with high quality, rare variants at <1% frequency and miRNAs with high quality, rare variants at <5% frequency. (.xlsx, 58 KB)
