# Supplementary material for: Whole-Genome Sequencing Suggests Schizophrenia Risk Mechanisms in Humans with 22q11.2 Deletion Syndrome
Source: G3 (Bethesda). 2015 Sep 16;5(11):2453–61. doi: 10.1534/g3.115.021345 (PMC4632064; doi:10.1534/g3.115.021345)
Supplement: Supporting Information [file supp_g3.115.021345_021345SI.pdf]

G3 · Genes | Genomes | Genetics

# Whole-genome sequencing suggests schizophrenia risk mechanisms in humans with 22q11.2 deletion syndrome

Daniele Merico[1*], Mehdi Zarrei[1*], Gregory Costain[2,3], Lucas Ogura[2], Babak Alipanahi[4], Matthew J. Gazzellone[1], Nancy J. Butcher[2], **Bhooma Thiruvahindrapuram[1], Thomas Nalpathamkalam[1],** Eva W. C. Chow[2,5], Danielle M. Andrade[6,7], Brendan J. Frey[4], Christian R. Marshall[1], Stephen W. Scherer[1,8], and Anne S. Bassett[2,5,9-11]

[1]The Centre for Applied Genomics and Program in Genetics and Genome Biology, The Hospital for Sick Children, Toronto, Ontario, Canada

[2]Clinical Genetics Research Program, Centre for Addiction and Mental Health, Toronto, Ontario, Canada

[3]Medical Genetics Residency Training Program, University of Toronto, Toronto, Ontario, Canada

[4]Department of Electrical and Computer Engineering, University of Toronto, Toronto, Ontario Canada

[5]Department of Psychiatry, University of Toronto, Toronto, Ontario, Canada

[6]Division of Neurology, Department of Medicine, University of Toronto, Toronto, Ontario, Canada

[7]Epilepsy Genetics Program, Toronto Western Hospital, University Health Network and University of Toronto, Toronto, Ontario, Canada

[8]McLaughlin Centre and Department of Molecular Genetics, University of Toronto, Toronto, Ontario, Canada

[9]Department of Psychiatry, and Toronto General Research Institute, University Health Network, Toronto, Ontario, Canada

[10]Campbell Family Mental Health Research Institute, Centre for Addiction and Mental Health, Toronto, Ontario, Canada

[11]The Dalglish Family Hearts and Minds Clinic for 22q11.2 Deletion Syndrome, Toronto General Hospital, University Health Network, Toronto, Ontario, Canada

**Corresponding author:** Anne S. Bassett, Centre for Addiction and Mental Health, 33 Russell Street, Room 1100, Toronto, Ontario, Canada M5S 2S1, Phone: (416) 535-8501 x32731, Fax: (416) 535-7199, E-mail: anne.bassett@utoronto.ca

*These authors contributed equally to this work.

**DOI: 10.1534/g3.115.021345**

Figure S1

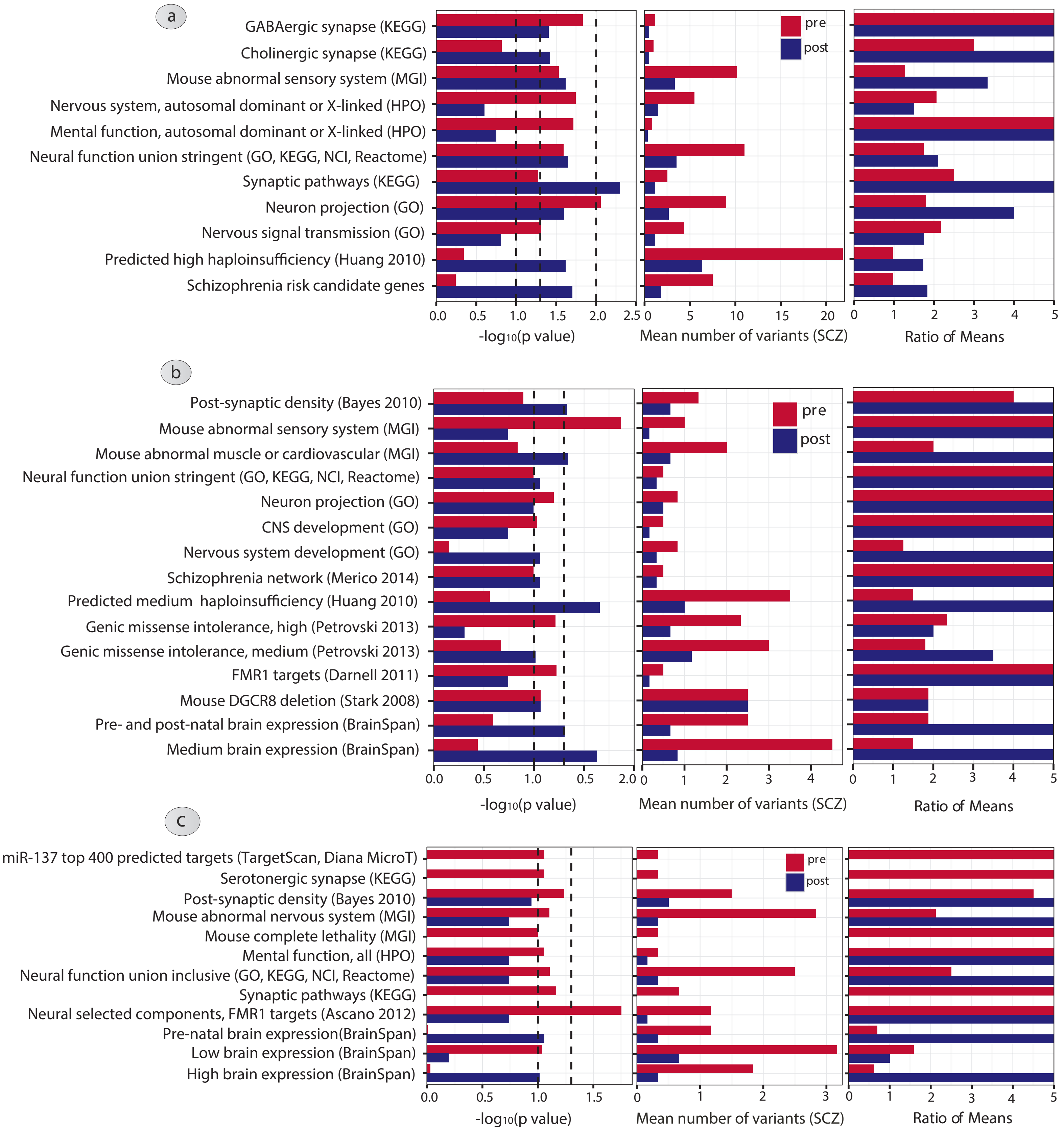


D. Merico et al.

**Figure S1** Selected gene-sets with a higher burden of (a) damaging missense, (b) loss of function (LoF), and (c) splicing regulatory variants in those with 22q11.2DS and schizophrenia (SCZ1-SCZ6) compared with those with 22q11.2DS and no psychotic disorder (NP1-NP3). Results are shown for gene-sets both pre- and post-intersection with the *DGCR8*-related gene-set. Nominal (one-sided t-test) p-values were calculated using percent values (means corrected for total number of all variants of that type per subject). Only gene-sets with $p<0.05$ for missense variants, or $p<0.10$ for LoF variants or splicing regulatory variants, either pre- or post-intersection with the *DGCR8*-related gene-set, are shown. The dashed vertical lines in the leftmost bar graphs indicate p-value thresholds (from left to right) of 0.10, 0.05, and 0.01. The bar graphs in the centre display the mean number of variants in each gene-set in the schizophrenia group. The rightmost bar graphs show the between-group ratios of the mean absolute variant counts, as a measure of the burden effect size. Where the mean ratio is not calculable (i.e., infinity, with no variants in the non-psychotic group), the bars terminate at 5. See text and Table 2 for additional details. All high quality, rare variants contributing to the results are reported in Table S1. For source, total size of each gene-set, and gene overlap between gene-sets see Table S2; for burden analysis results for all gene-sets, see Table S3. SCZ = Schizophrenia subgroup of 22q11.2DS subjects; GO = Gene Ontology; KEGG = Kyoto Encyclopedia of Genes and Genomes; MGI = Mouse Genome Informatics; HPO = Human Phenotype Ontology; NCI = National Cancer Institute

Figure S2

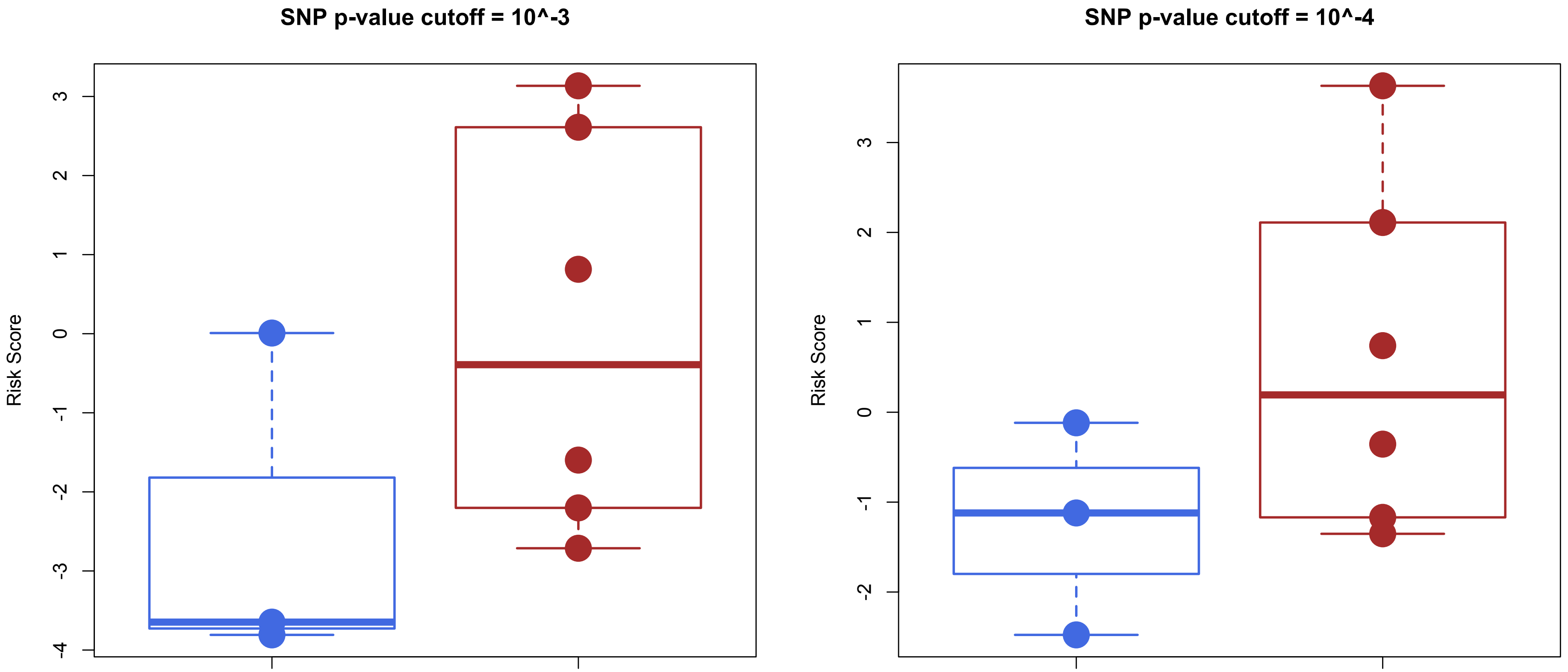
SNP p-value cutoff = 10^-3
Risk Score
3
2
1
0
-1
-2
-3
-4
SNP p-value cutoff = 10^-4
Risk Score
3
2
1
0
-1
-2

**Figure S2.** Distribution boxplots of subjects' polygenic risk scores for the schizophrenia (brown outline) and non-psychotic (blue outline) groups, using the SNPs at association p-value cutoff of 0.001 (10^-3) and 0.0001 (10^-4). Please see Methods for details.

**Whole-genome sequencing reveals schizophrenia risk mechanisms in humans with 22q11.2 deletion syndrome**

# File S1

## SUPPLEMENTAL METHODS

Methods for whole-genome sequencing (WGS) and annotation of variants for this study were based on those used for a WGS study of autism (Yuen *et al.* 2015).

### Whole-genome sequencing

Genomic DNA extracted from blood was assessed for quality by PicoGreen and gel electrophoresis, and then sequenced by Complete Genomics (Mountain View, CA) (Drmanac *et al.* 2010). At least 10 μg of non-degraded DNA was provided for WGS. Complete Genomics performed additional quality controls, including DNA quality assessment, sex checking, and comparison of samples with results from a 96-SNP genotyping assay to prevent sample mix-ups. The Complete Genomics Analysis Platform employs high-density DNA nanoarrays that are populated with 35-base, mate-paired reads, generated from the ends of approximately 500 bp genomic fragments biochemically engineered into DNA nanoballs (Drmanac *et al.* 2010). Base identification was performed using a non-sequential, unchained read technology known as combinatorial probe-anchor ligation (Drmanac *et al.* 2010). The genome coverage per sample was on average 98.95% (98.81-99.10%) at depth ≥ 5X and 97.65% (97.30-98.15%) at depth ≥10X. Likewise, 95.6% and 74.8% of the exome was covered with at least 20X and 40X sequence depth, respectively

### Annotation of rare sequence-based variants

***Quality:*** Single nucleotide variants (SNVs) and small insertions/deletions (indels) were called by the proprietary Complete Genomics pipeline (pipeline and assembly version 2.2) (Carnevali *et al.* 2012). Based on our prior experience with this platform (Yuen *et al.* 2015), we defined more stringent quality filters as follows: (i) sequencing depth ≥5, (ii) allele quality VQHIGH for both alleles, (iii) alternate allele fraction ≥80% for haploid and homozygous variants or ≥30% for heterozygous variants, (iv) equal allele fraction (EAF) allele score ≥40 for heterozygous variants or ≥20 for homozygous variants, and (v) called ploidy =1 for haploid or hemizygous variants and =2 for other variants. Only the higher quality variants meeting these additional criteria were used for analyses in this study.

Variants were annotated for rarity and category using a custom pipeline based on ANNOVAR (November 2014) (Wang *et al.* 2010).

***Rarity:*** Both publicly available and internal databases were used for annotating allele frequency and defining rare variants: (i) 1000 Genomes (Genomes Project *et al.* 2012), (ii) NHLBI Exome Sequencing Project (NHLBI-ESP) (Fu *et al.* 2013), (iii) Exome Aggregation Consortium (ExAC; http://exac.broadinstitute.org/), and (iv) internal Complete Genomics control databases. We defined rare variants as those not exceeding the 1% alternate allele frequency threshold in each of these four databases, considering the full control population cohort as well as each major ethnic subgroup (1000 Genomes: Caucasian, African, Latin American, East Asian, South Asian; NHLBI-ESP: Caucasian, African-American; ExAC: Caucasian (not Finnish), Finnish, African, Latin American, East Asian, South Asian, other).

***Category and deleteriousness:*** RefSeq gene models were used to determine the variant category (e.g., coding exonic, UTR, intronic) and effect on gene products.

Loss of function (LoF), damaging missense, and splicing regulatory variants were collectively termed “coding variants”. The greatest impact was attributed to bona fide complete loss of function (LoF) variants (Table S1), consisting of stop-gain/nonsense, frameshift and core splice site altering variants (where a core splice site corresponds to the 2 intronic bp adjacent to an intron-exon junction).

To score the impact of missense variants we employed established predictors: (i) SIFT (Ng and Henikoff 2001), (ii) PolyPhen2 (Adzhubei *et al.* 2010), (iii) Mutation Assessor (Reva *et al.* 2007), (iv) MutationTaster2 (Schwarz *et al.* 2014), (v) CADD (Kircher *et al.* 2014), and (vi) the genomic conservation indexes PhyloP and phastCons for placental mammals and 100 vertebrates (Pollard *et al.* 2010). Missense variants were labelled as “damaging” when they met at least four of these seven criteria: (i) SIFT ≤0.05, (ii) PolyPhen2 ≥0.90, (iii) Mutation Assessor ≥1.9, (iv) MutationTaster2 score >0.5, (v) CADD Phred score ≥15, (vi) placental mammal PhyloP ≥2.3, and (vii) vertebrate PhyloP ≥4. Damaging missense variants (Table S1) were used for the main gene-set burden analysis.

Finally, we used a recently published method (Xiong *et al.* 2015) to identify exonic and intronic SNVs with predicted regulatory effect on splicing. For this study we focused on variants predicted to decrease exon inclusion in spliced transcripts at a stringent level (dPSI ≤-5, corresponding to a five point decrease of percentage exon inclusion for the variant allele compared to the reference sequence) (Table S1); results involving less stringent levels or

increased exon inclusion can be more difficult to interpret. Variants already classified as LoF or damaging missense were not included in this splicing regulatory category.

**Curation of schizophrenia-related gene-sets**

In order to assess the burden of rare coding variants (LoF, damaging missense, and splicing regulatory), we curated diverse database resources and the literature to compile sets of genes relevant to schizophrenia and related neuropsychiatric/neurodevelopmental disorders (Yuen *et al.* 2015; Costain *et al.* 2013; Engchuan *et al.* 2015; Merico *et al.* 2014). We also assessed large, generally more non-specific gene-sets (23 gene-sets with >2,000 genes, including one with 15,944 human well-characterized genes, four with genes having predictions related to haploinsufficiency, and four with genes having predictions related to intolerance to nonsynonymous variation). Only three of these gene-sets were considered neurofunctional, in addition to the seven BrainSpan gene-sets noted below. Last, we curated gene-sets for diverse non-neurological organ system functions in mice (seven gene-sets, including three with >2,000 genes included in the above count of 23).

Gene-sets included in the burden analyses fell into one of three size-based categories: large (>2,000 genes; generally non-specific gene-sets), small (≤200 genes; generally nested subsets of other gene-sets), and the remainder. The latter category comprised most of the neurofunctional gene-sets relevant to schizophrenia, and accounts for the majority of results presented in the text and Table 2.

The neurofunctional gene-sets included:

(i) genes annotated for general neural functions and pathways, based on Gene Ontology (GO) and pathway databases (Biocarta, KEGG, NCI, Reactome) (8 gene-sets, including 1 large gene-set with a more stringent, nested counterpart);

(ii) synaptic genes (2 gene-sets (GO and KEGG), and 3 small nested subsets) and genes encoding proteins found in the post-synaptic density (1 gene-set) (Bayes *et al.* 2011);

(iii) other neuronal components previously implicated in schizophrenia, including genes regulated by the *FMR1* protein product (2 gene-sets) (Darnell *et al.* 2011; Ascano *et al.* 2012), the *DISC1* interactome (3 gene-sets: top 100, 50, 25), and other selected components (3 small gene-sets, each with <65 genes (Kirov *et al.* 2012; Purcell *et al.* 2014));

(iv) genes implicated in human neurodevelopmental and psychotic conditions, based on the Human Phenotype Ontology (HPO) (6 gene-sets, including 3 nested subsets restricted to autosomal dominant and X-linked mechanisms);

(v) orthologs of genes associated with neurodevelopmental and abnormal behavior phenotypes in mice, as annotated in the Mouse Genome Informatics (MGI) database (3 gene-sets, including 2 large gene-sets);

(vi) genes with nonsynonymous *de novo* mutations in WES studies of schizophrenia (Girard *et al.* 2011; Xu *et al.* 2012; Gulsuner *et al.* 2013; Fromer *et al.* 2014; McCarthy *et al.* 2014; Guipponi *et al.* 2014) (2 gene-sets, one a nested subset with just the Fromer et al. 2014 results), and genes in a proposed schizophrenia network (1 gene-set);

(vii) genes expressed in the human brain, grouped by expression level (4 large gene-sets) and developmental stage (3 large gene-sets), based on the BrainSpan expression atlas (www.brainspan.org);

(viii) for a related study using the same WGS data, gene-sets designed to assess Parkinson's disease/abnormal extrapyramidal functions (6 gene-sets, including 3 small sets (<200 genes)); and,

(ix) predicted targets of two microRNAs (miRNAs) previously implicated in schizophrenia (top 800, top 400, and top 200 gene targets for each of miR-137 and miR-185, for a total of 6 gene-sets).

To specifically test our *DGCR8*/miRNA hypothesis, we used the large gene-set comprising 3,558 genes differentially expressed in a mouse model of *DGCR8* haploinsufficiency (Stark *et al.* 2008; Merico *et al.* 2014). We tested for enrichment in this large, more non-specific *DGCR8* gene-set directly. More importantly, we also intersected this gene-set with the other gene-sets to assess the impact of this mechanism on burden, particularly burden related to neurofunctional gene-sets.

The gene-sets used and the intersection between the gene-sets with respect to their gene content, including effects of the *DGCR8* gene-set restriction, are provided in Table S2.

**Gene-set burden analyses**

We defined gene-set burden as the percentage of coding variants in a given category (e.g., damaging missense) that map to genes from the gene-set being tested. This ensures robustness to inter-individual differences in the total number of rare damaging variants, which may be due to technical or subethnic confounders. Differences between the schizophrenia and non-psychotic groups were assessed using a one-tailed Student's t-test. Because of the small sample size, we used an inclusive nominal *p*-value threshold ($p<0.10$ for LoF and splicing regulatory variants, $p<0.05$ for missense variants), though we display only $p<0.05$ results in Table 2. All results will

have to be confirmed by larger studies. To estimate the burden effect size, we calculated the between-group ratio of the mean absolute variant count (Hu *et al.* 2009). We tested each coding-related SNV category (LoF, damaging missense, splicing regulatory) separately because we expected different effect sizes and prevalence in the schizophrenia and non-psychotic groups (Tables S3). We used a multivariate two-sample Hotelling's T-Square test (Hotelling 1931) to test the joint burden of these three variant categories, in the subset of gene-sets having a higher absolute variant count in the schizophrenia group and reaching nominal p-value thresholds for at least two categories.

**Power calculations**

For coding variants, we performed power calculations for the gene-set burden test (schizophrenia – non-psychotic difference of the gene-set variant percentages, one-tailed Student's t-test). We selected four representative gene-sets showing significant enrichment for at least one of the categories of variants, and used Cohen's d to express the effect size estimates based on this pilot data-set. We calculated the power of the burden test using N = (25, 50, 100, 500) x 2 (Table S5).

**Assessment of copy number and other structural variants**

We evaluated copy number variations (CNVs) and other structural variants (SVs) using a rigorous detection process, as described elsewhere (Yuen *et al.* 2015). Deletions and duplications were analyzed separately. CNV calls were excluded if they overlapped segmental duplications by more than 80%. Rare CNVs were defined as those: (i) absent from the Complete Genomics Diversity Panel and (ii) present at a frequency of ≤0.05 in the parents of probands with autism spectrum disorders genotyped in our previous WGS study (Yuen *et al.* 2015), using 50% reciprocal overlap criteria (Pinto *et al.* 2014; Zarrei *et al.* 2015; Costain *et al.* 2013; Silversides *et*

*al.* 2012). CNVs were further adjudicated for rarity using the Database of Genomic Variants (http://dgv.tcag.ca) (MacDonald *et al.* 2014). Similarly, other SVs were required to have a minimum mate pair count of 20, and were retained only if they were absent from the Complete Genomics Diversity Panel and the SV Baseline Genome Dataset.

All subjects were confirmed to have 22q11.2 deletions (Table 1). Of the remaining variants, only rare CNVs and SVs that overlapped at least one coding gene exon of a RefSeq gene with known neuronal function were considered in this study. We had previously genotyped all samples on high-resolution microarray platforms for the detection of CNVs [(Bassett *et al.* 2008) and data not shown].

**Variants in non-coding RNA genes**

We mapped all miRNAs included in mirBase v20 (Griffiths-Jones 2004) and all long intergenic non-coding RNAs (lincRNAs) in the Broad catalogue (Cabili *et al.* 2011). (Griffiths-Jones 2004; Cabili *et al.* 2011)Rare, high quality sequence variants in these non-coding RNAs were prioritized using the following criteria: (i) conservation at the nucleotide level (PhyloP), and (ii) overlap with conserved elements (PhastCons) (Siepel *et al.* 2005). Regarding the former, we required CADD_phred ≥15, and either phylopPMam_avg ≥1.75 or phylopVert100_avg ≥2.25. We annotated miRNA variants using both primary and mature miRNA transcripts. Table S7 lists lincRNAs and miRNAs with high quality rare variants.

We tested the burden of rare variants in the set of all lincRNA (n=4,273) (Cabili *et al.* 2011), as well as in two lincRNA subsets with higher conservation (PhastCons; (Siepel *et al.* 2005)): the first subset included any lincRNA overlapping at least one PhastCons conserved element

(n=2,082), while the second subset consisted of lincRNA with >26% of their sequence overlapped by PhastCons elements (n=222, corresponding to the top 10% conserved lincRNA). We also tested the burden for the brain-expressed lincRNA subset (206 genes) (Cabili *et al.* 2011) (Tables S6 and S7).

We compiled four conserved miRNA sets for the current analysis (Table S7). The first set comprised miRNA that were overlapped by rare CNVs in two or more unrelated adult cases with schizophrenia in a cohort of 420 subjects without 22q11.2 deletions (n=20 miRNAs) (Warnica *et al.* 2015). We additionally compiled three sets using the BrainSpan expression data (Hu *et al.* 2011). We defined a set of brain expressed miRNA with all genes having a minimum of 30 reads and expressed at a minimum of two different brain regions or subjects (n=542). We further defined a set of well-expressed miRNA in brain with a minimum of 350 reads that are expressed in minimum of three brain regions or subjects (n=293). We defined miRNAs with rare variants at the <1% frequency cutoff as described above. We also used a relaxed frequency threshold (<5%) to investigate additional miRNA because of the rarity of these genes in the genome compared with lincRNA and protein-coding genes.

## LITERATURE CITED

Adzhubei, I. A., S. Schmidt, L. Peshkin, V. E. Ramensky, A. Gerasimova *et al.*, 2010 A method and server for predicting damaging missense mutations. *Nat. Methods.* 7: 248-249.

Ascano, M., Jr., N. Mukherjee, P. Bandaru, J. B. Miller, J. D. Nusbaum *et al.*, 2012 FMRP targets distinct mRNA sequence elements to regulate protein expression. *Nature.* 492: 382-386.

Bassett, A. S., C. R. Marshall, A. C. Lionel, E. W. Chow, and S. W. Scherer, 2008 Copy number variations and risk for schizophrenia in 22q11.2 deletion syndrome. *Hum. Mol. Genet.* 17: 4045-4053.

Bayes, A., L. N. van de Lagemaat, M. O. Collins, M. D. Croning, I. R. Whittle *et al.*, 2011 Characterization of the proteome, diseases and evolution of the human postsynaptic density. *Nat. Neurosci.* 14: 19-21.

Cabili, M. N., C. Trapnell, L. Goff, M. Koziol, B. Tazon-Vega *et al.*, 2011 Integrative annotation of human large intergenic noncoding RNAs reveals global properties and specific subclasses. *Genes Dev.* 25: 1915-1927.

Carnevali, P., J. Baccash, A. L. Halpern, I. Nazarenko, G. B. Nilsen *et al.*, 2012 Computational techniques for human genome resequencing using mated gapped reads. *J. Comp. Biol.* 19: 279-292.

Costain, G., A. C. Lionel, D. Merico, P. Forsythe, K. Russell *et al.*, 2013 Pathogenic rare copy number variants in community-based schizophrenia suggest a potential role for clinical microarrays. *Hum. Mol. Genet.* 22: 4485–4501.

Darnell, J. C., S. J. Van Driesche, C. Zhang, K. Y. Hung, A. Mele *et al.*, 2011 FMRP stalls ribosomal translocation on mRNAs linked to synaptic function and autism. *Cell.* 146: 247-261.

Drmanac, R., A. B. Sparks, M. J. Callow, A. L. Halpern, N. L. Burns *et al.*, 2010 Human genome sequencing using unchained base reads on self-assembling DNA nanoarrays. *Science.* 327: 78-81.

Engchuan, W., K. Dhindsa, A. C. Lionel, S. W. Scherer, J. H. Chan *et al.*, 2015 Performance of case-control rare copy number variation annotation in classification of autism. *BMC Med. Genomics.* 8: 1-10.

Fromer, M., A. J. Pocklington, D. H. Kavanagh, H. J. Williams, S. Dwyer *et al.*, 2014 De novo mutations in schizophrenia implicate synaptic networks. *Nature.* 506: 179-184.

Fu, W., T. D. O'Connor, G. Jun, H. M. Kang, G. Abecasis *et al.*, 2013 Analysis of 6,515 exomes reveals the recent origin of most human protein-coding variants. *Nature.* 493: 216-220.

Genomes Project, C., G. R. Abecasis, A. Auton, L. D. Brooks, M. A. DePristo *et al.*, 2012 An integrated map of genetic variation from 1,092 human genomes. *Nature.* 491: 56-65.

Girard, S. L., J. Gauthier, A. Noreau, L. Xiong, S. Zhou *et al.*, 2011 Increased exonic de novo mutation rate in individuals with schizophrenia. *Nat. Genet.* 43: 860-863.

Griffiths-Jones, S., 2004 The microRNA Registry. *Nucleic Acids Res.* 32: D109-111.

Guipponi, M., F. A. Santoni, V. Setola, C. Gehrig, M. Rotharmel *et al.*, 2014 Exome sequencing in 53 sporadic cases of schizophrenia identifies 18 putative candidate genes. *PLoS One.* 9: e112745.

Gulsuner, S., T. Walsh, A. C. Watts, M. K. Lee, A. M. Thornton *et al.*, 2013 Spatial and temporal mapping of de novo mutations in schizophrenia to a fetal prefrontal cortical network. *Cell.* 154: 518-529.

Hotelling, H., 1931 The generalization of Student's ratio. *Ann Math Stat.* 2: 360-378.

Hu, H. Y., S. Guo, J. Xi, Z. Yan, N. Fu *et al.*, 2011 MicroRNA expression and regulation in human, chimpanzee, and macaque brains. *PLoS Genet.* 7: e1002327.

Hu, P., C. M. Greenwood, and J. Beyene, 2009 Using the ratio of means as the effect size measure in combining results of microarray experiments. *BMC Syst. Biol.* 3: 106.

Kircher, M., D. M. Witten, P. Jain, B. J. O'Roak, G. M. Cooper *et al.*, 2014 A general framework for estimating the relative pathogenicity of human genetic variants. *Nat. Genet.*

Kirov, G., A. J. Pocklington, P. Holmans, D. Ivanov, M. Ikeda *et al.*, 2012 De novo CNV analysis implicates specific abnormalities of postsynaptic signalling complexes in the pathogenesis of schizophrenia. *Mol. Psychiatry.* 17: 142-153.

MacDonald, J. R., R. Ziman, R. K. Yuen, L. Feuk, and S. W. Scherer, 2014 The Database of Genomic Variants: a curated collection of structural variation in the human genome. *Nucleic Acids Res.* 42: D986-992.

McCarthy, S. E., J. Gillis, M. Kramer, J. Lihm, S. Yoon *et al.*, 2014 De novo mutations in schizophrenia implicate chromatin remodeling and support a genetic overlap with autism and intellectual disability. *Mol. Psychiatry.* 19: 652-658.

Merico, D., G. Costain, N. J. Butcher, W. Warnica, L. Ogura *et al.*, 2014 MicroRNA dysregulation, gene networks and risk for schizophrenia in 22q11.2 deletion syndrome. *Front. Neurol.* 5: 238.

Ng, P. C., and S. Henikoff, 2001 Predicting deleterious amino acid substitutions. *Genome Res.* 11: 863-874.

Pinto, D., E. Delaby, D. Merico, M. Barbosa, A. Merikangas *et al.*, 2014 Convergence of genes and cellular pathways dysregulated in autism spectrum disorders. *Am. J. Hum. Genet.* 94: 677-694.

Pollard, K. S., M. J. Hubisz, K. R. Rosenbloom, and A. Siepel, 2010 Detection of nonneutral substitution rates on mammalian phylogenies. *Genome Res.* 20: 110-121.

Purcell, S. M., J. L. Moran, M. Fromer, D. Ruderfer, N. Solovieff *et al.*, 2014 A polygenic burden of rare disruptive mutations in schizophrenia. *Nature.* 506: 185-190.

Reva, B., Y. Antipin, and C. Sander, 2007 Determinants of protein function revealed by combinatorial entropy optimization. *Genome Biol.* 8: R232.

Schwarz, J. M., D. N. Cooper, M. Schuelke, and D. Seelow, 2014 MutationTaster2: mutation prediction for the deep-sequencing age. *Nat. Methods.* 11: 361-362.

Siepel, A., G. Bejerano, J. S. Pedersen, A. S. Hinrichs, M. Hou *et al.*, 2005 Evolutionarily conserved elements in vertebrate, insect, worm, and yeast genomes. *Genome Res.* 15: 1034-1050.

Silversides, C. K., A. C. Lionel, G. Costain, D. Merico, O. Migita *et al.*, 2012 Rare copy number variations in adults with tetralogy of Fallot implicate novel risk gene pathways. *PLoS Genetics.* 8: e1002843.

Stark, K. L., B. Xu, A. Bagchi, W. S. Lai, H. Liu *et al.*, 2008 Altered brain microRNA biogenesis contributes to phenotypic deficits in a 22q11-deletion mouse model. *Nat. Genet.* 40: 751-760.

Wang, K., M. Li, and H. Hakonarson, 2010 ANNOVAR: functional annotation of genetic variants from high-throughput sequencing data. *Nucleic Acids Res.* 38: e164.

Warnica, W., D. Merico, G. Costain, S. E. Alfred, J. Wei *et al.*, 2015 Copy number variable microRNAs in schizophrenia and their neurodevelopmental gene targets. *Biol. Psychiatry.* 77: 158-166.

Xiong, H. Y., B. Alipanahi, L. J. Lee, H. Bretschneider, D. Merico *et al.*, 2015 RNA splicing. The human splicing code reveals new insights into the genetic determinants of disease. *Science.* 347: 1254806.

Xu, B., I. Ionita-Laza, J. L. Roos, B. Boone, S. Woodrick *et al.*, 2012 De novo gene mutations highlight patterns of genetic and neural complexity in schizophrenia. *Nat. Genet.* 44: 1365-1369.

Yuen, R. K., B. Thiruvahindrapuram, D. Merico, S. Walker, K. Tammimies *et al.*, 2015 Whole-genome sequencing of quartet families with autism spectrum disorder. *Nat. Med.* 185-191.

Zarrei, M., J. R. MacDonald, D. Merico, and S. W. Scherer, 2015 A copy number variation map of the human genome. *Nat. Rev. Genet.* 16: 172-183.

**Tables S1-S6**

**Available for download as Excel files at www.g3journal.org/lookup/suppl/doi:10.1534/g3.115.021345/-/DC1**

**Table S1:** High quality, rare (<1% frequency) coding variants in subjects with 22q11.2DS used in the gene-set burden analyses. Variants in the intact chromosome 22q11.2 region and on the X chromosome were examined separately. Variant types include: loss of function, damaging missense, and splicing regulatory (see text and Supplemental Methods for details). All variants are annotated for gene-sets (details of which are in Table S2).

**Table S2.** Source, size, and pair-wise intersection of gene-sets used in the burden analyses, by number of genes and percent overlap.

**Table S3.** Details of burden analyses for each of damaging missense, loss of function, and splicing regulatory variant categories, comparing subjects with 22q11.2DS and schizophrenia to subjects with 22q11.2DS and no psychotic disorder. See text and Supplemental Methods for details.

**Table S4.** Most recurrent splicing regulatory predictive features detected in this study (see Supplemental Methods and (Xiong *et al.* 2015) for details).

**Table S5.** Details of power calculation for enrichment in selected gene-sets based on the results of the current study.

**Table S6**. Details of burden analyses for lincRNAs with rare conserved variants.

**Table S7.** Details of lincRNAs with high quality, rare variants at <1% frequency and miRNAs with high quality, rare variants at <5% frequency.
