# Supplementary material for: Whole-Genome Sequencing Suggests Schizophrenia Risk Mechanisms in Humans with 22q11.2 Deletion Syndrome
Source: G3 (Bethesda). 2015 Sep 16;5(11):2453–61. doi: 10.1534/g3.115.021345 (PMC4632064; doi:10.1534/g3.115.021345)
Supplement: Supporting Information [file supp_g3.115.021345_FigureS1.pdf]

Figure S1

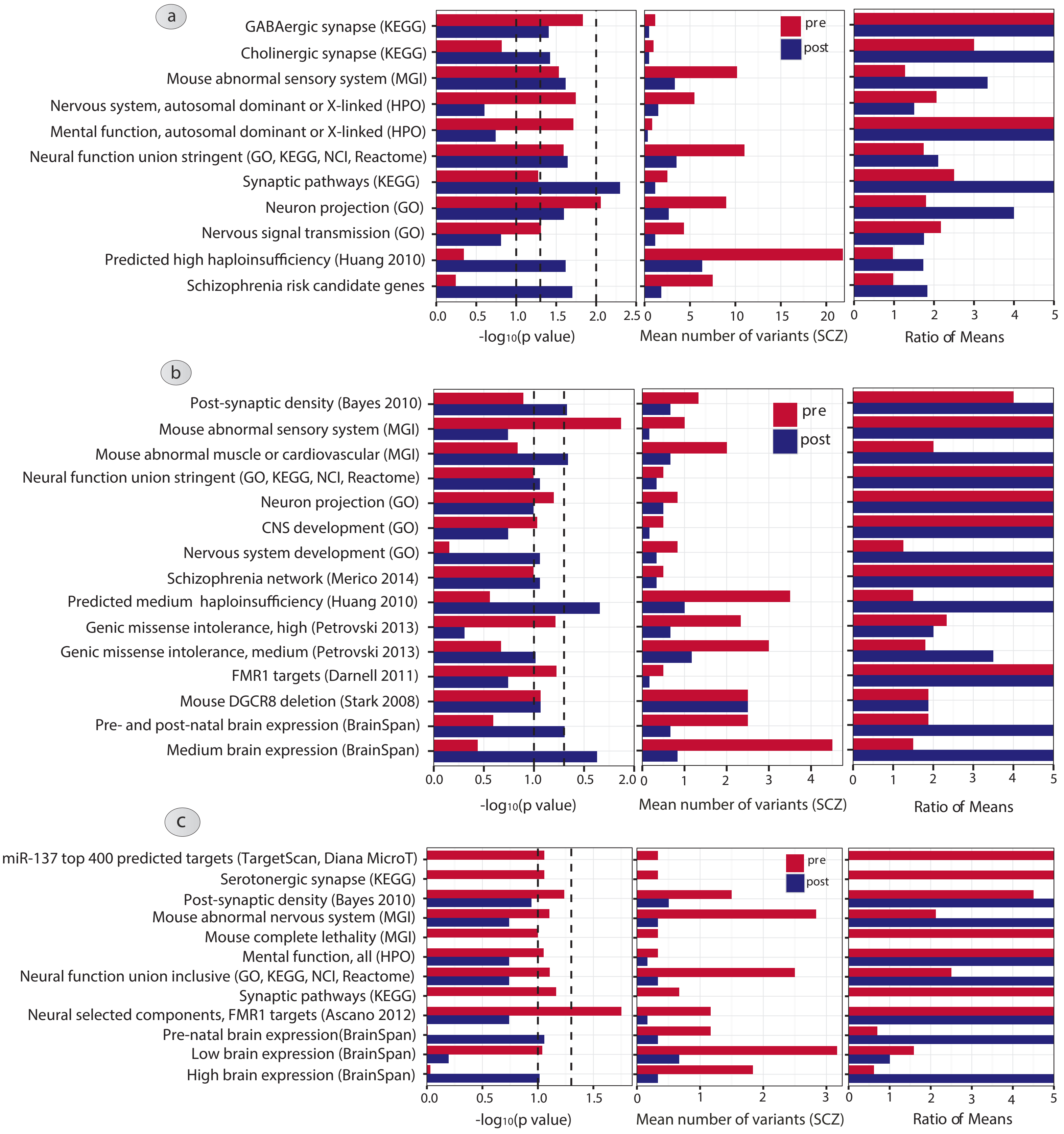

D. Merico et al.

Figure S1 Selected gene-sets with a higher burden of (a) damaging missense, (b) loss of function (LoF), and (c) splicing regulatory variants in those with 22q11.2DS and schizophrenia (SCZ1-SCZ6) compared with those with 22q11.2DS and no psychotic disorder (NP1-NP3). Results are shown for gene-sets both pre- and post-intersection with the *DGCR8*-related gene-set. Nominal (one-sided t-test) p-values were calculated using percent values (means corrected for total number of all variants of that type per subject). Only gene-sets with $p<0.05$ for missense variants, or $p<0.10$ for LoF variants or splicing regulatory variants, either pre- or post-intersection with the *DGCR8*-related gene-set, are shown. The dashed vertical lines in the leftmost bar graphs indicate p-value thresholds (from left to right) of 0.10, 0.05, and 0.01. The bar graphs in the centre display the mean number of variants in each gene-set in the schizophrenia group. The rightmost bar graphs show the between-group ratios of the mean absolute variant counts, as a measure of the burden effect size. Where the mean ratio is not calculable (i.e., infinity, with no variants in the non-psychotic group), the bars terminate at 5. See text and Table 2 for additional details. All high quality, rare variants contributing to the results are reported in Table S1. For source, total size of each gene-set, and gene overlap between gene-sets see Table S2; for burden analysis results for all gene-sets, see Table S3. SCZ = Schizophrenia subgroup of 22q11.2DS subjects; GO = Gene Ontology; KEGG = Kyoto Encyclopedia of Genes and Genomes; MGI = Mouse Genome Informatics; HPO = Human Phenotype Ontology; NCI = National Cancer Institute
