# Supplementary material for: Whole-Genome Sequencing Suggests Schizophrenia Risk Mechanisms in Humans with 22q11.2 Deletion Syndrome
Source: G3 (Bethesda). 2015 Sep 16;5(11):2453–61. doi: 10.1534/g3.115.021345 (PMC4632064; doi:10.1534/g3.115.021345)
Supplement: Supporting Information [file supp_g3.115.021345_FigureS2.pdf]

Figure S2

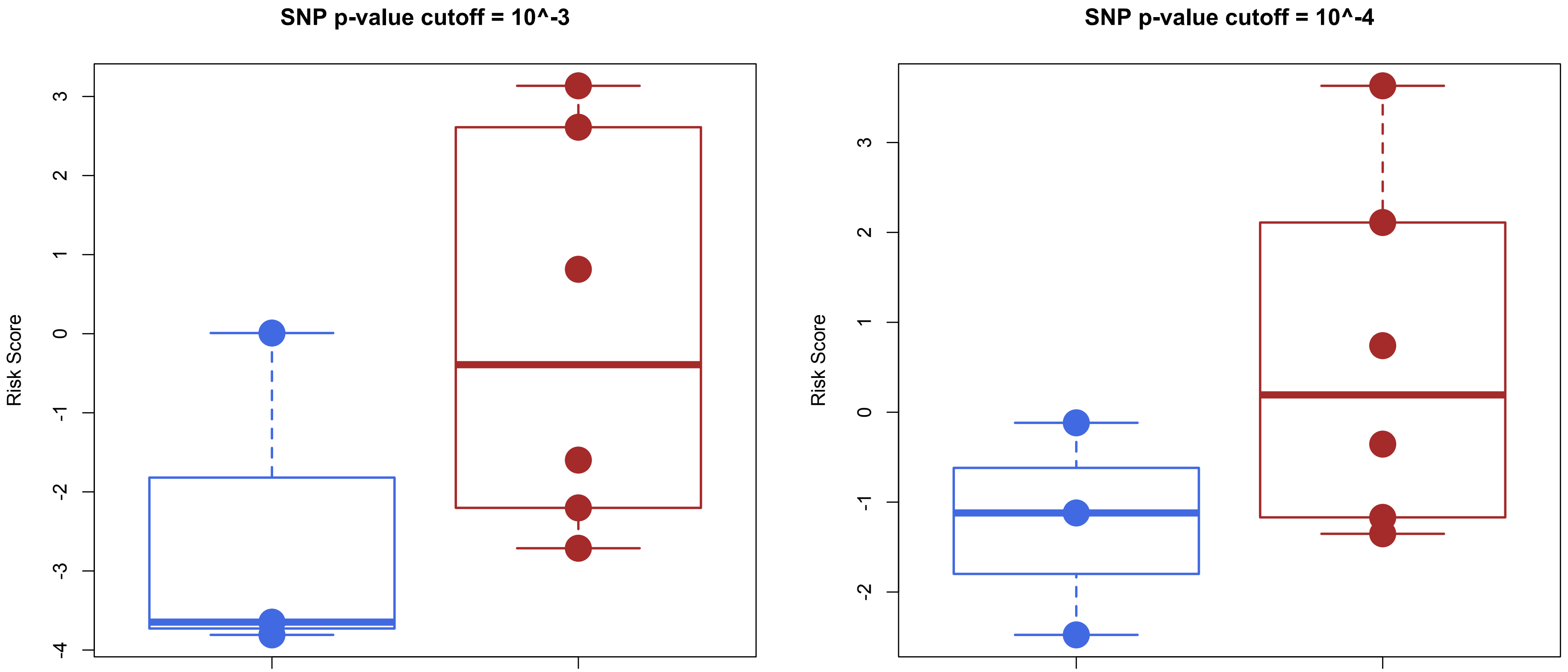

D. Merico et al.

Figure S2. Distribution boxplots of subjects' polygenic risk scores for the schizophrenia (brown outline) and non-psychotic (blue outline) groups, using the SNPs at association p-value cutoff of 0.001 (10^-3) and 0.0001 (10^-4). Please see Methods for details.
